# Bypassing hazard of housekeeping genes: their evaluation in rat granule neurons treated with cerebrospinal fluid of multiple sclerosis subjects

**DOI:** 10.3389/fncel.2015.00375

**Published:** 2015-09-23

**Authors:** Deepali Mathur, Juan R. Urena-Peralta, Gerardo Lopez-Rodas, Bonaventura Casanova, Francisco Coret-Ferrer, Maria Burgal-Marti

**Affiliations:** ^1^Department of Functional Biology, University of ValenciaValencia, Spain; ^2^Multiple Sclerosis Laboratory, Department of Biomedicine, Prince Felipe Research CenterValencia, Spain; ^3^Department of Biochemistry and Molecular Biology, University of Valencia and INCLIVA Biomedical Research InstituteValencia, Spain; ^4^CSUR-Esclerosi Múltiple, Hospital Universitari i Politècnic La Fe, Unitat Mixta d’Esclerosi Múltiple i Neurorregeneració de l’IIS-La FeValència, Spain; ^5^Hospital Clínico, Universitario de ValenciaValencia, Spain

**Keywords:** housekeeping genes, multiple sclerosis, normalization, *GeNorm*, *NormFinder*

## Abstract

Gene expression studies employing real-time PCR has become an intrinsic part of biomedical research. Appropriate normalization of target gene transcript(s) based on stably expressed housekeeping genes is crucial in individual experimental conditions to obtain accurate results. In multiple sclerosis (MS), several gene expression studies have been undertaken, however, the suitability of housekeeping genes to express stably in this disease is not yet explored. Recent research suggests that their expression level may vary under different experimental conditions. Hence it is indispensible to evaluate their expression stability to accurately normalize target gene transcripts. The present study aims to evaluate the expression stability of seven housekeeping genes in rat granule neurons treated with cerebrospinal fluid of MS patients. The selected reference genes were quantified by real time PCR and their expression stability was assessed using *GeNorm* and *NormFinder* algorithms. *GeNorm* identified transferrin receptor (*Tfrc*) and microglobulin beta-2 (*B2m*) the most stable genes followed by ribosomal protein L19 (*Rpl19*) whereas β-actin (*ActB*) and glyceraldehyde-3-phosphate-dehydrogenase (*Gapdh*) the most fluctuated ones in these neurons. *NormFinder* identified *Tfrc* as the best invariable gene followed by *B2m* and *Rpl19*. *ActB* and *Gapdh* were the least stable genes as analyzed by *NormFinder* algorithm. Both methods reported *Tfrc* and *B2m* the most stably expressed genes and *Gapdh* the least stable one. Altogether our data demonstrate the significance of pre-validation of housekeeping genes for accurate normalization and indicates *Tfrc* and *B2m* as best endogenous controls in MS. *ActB* and *Gapdh* are not recommended in gene expression studies related to current one.

## Introduction

Techniques employed for calibrating gene expression are paramount in studies directed toward accurate analysis of transcriptomic profiles. Quantitative real time PCR (qRT-PCR) has gained significant momentum over the past decade to quantify gene expression profiles. Considering the utmost sensitivity and reliability of qRT-PCR, a careful selection of a constitutively expressed gene is required to account for variation in the amount and quality of starting RNA and cDNA synthesis efficiency. In general, the expression of target gene transcripts is normalized with an internal control, often referred to as a housekeeping gene. Housekeeping (HK) genes are endogenous controls that are required for the primary function of a cell hence their expression should be constant in all conditions. However, recent research has indicated that their expression may not necessarily be stable in all cells/tissues. A gene showing consistent expression in one condition may show unstable expression in another. Invariable expression of the so-called housekeeping genes has been observed during cellular development (Al-Bader and Al-Sarraf, 2005) and under distinct experimental conditions (Zhong and Simons, 1999; Hamalainen et al., 2001; Deindl et al., 2002; Glare et al., 2002; Torres et al., 2003; Radonic et al., 2004; Toegel et al., 2007; Gubern et al., 2009). Therefore it is essential to pre-validate the expression stability of reference genes to accurately normalize the gene expression data. It is recommended that more than one stably expressed gene should be used for precise normalization procedure (Zhong and Simons, 1999; Tricarico et al., 2002; Vandesompele et al., 2002; Ohl et al., 2005).

In this context, we aimed to evaluate the expression stability of seven commonly used housekeeping genes in cerebellar granule neurons (CGNs) treated with cerebrospinal fluid (CSF) from multiple sclerosis (MS) and neuromyelitis optica (NMO) patients. Axonal damage is widely accepted as a major cause of persistent functional disability in MS. Therefore to study primary neuronal damage independent of secondary damage, resulting from demyelination, we used primary cultures of unmyelinated CGNs as a cellular model and exposed them to CSF derived from MS patients. Prior to comprehending mechanisms involved in axonal degeneration–regeneration, it was first necessary to identify best stably expressed housekeeping genes that can be used to normalize target mRNA transcripts in our experimental system. We therefore used a xenogeneic system comprising of primary rat CGN cultures incubated with CSF from patients with MS or controls and investigated the stability of reference genes in these rat neuronal cells. Previous studies in similar xenogeneic models showed that treatment with human CSF resulted in neurotoxicity in culture, although the molecular mechanisms remained unknown (Xiao et al., 1996; Alcazar et al., 2000). Recently, Vidaurre et al. (2014) reported that ceramides present in CSF from patients with MS disturb neuronal bioenergetics in rat neuronal cultures.

Primary cultures of rat CGNs represent an excellent model to study almost every aspect of neurobiology. While neuronal cell lines have been very useful in the study of neuronal cell cultures, there are certain drawbacks they exhibit. These cell lines are derived from neuronal tumors and hence will show many important physiological differences with the cell type from which they were derived. For instance, the human SH-SY5Y cell line, was derived by subcloning from the parental metastatic bone tumor biopsy cell line SK-N-SH (Biedler et al., 1973). Therefore, it is prudent to use primary cultures because they are not tumor-derived and hence are more likely to exhibit the properties of neuronal cells *in vivo*. Furthermore, CGNs are small and the most numerous unmyelinated neurons, therefore we used primary cultures of rat CGNs as a cellular model and exposed them to diseased CSF to comprehend the pathophysiological mechanisms implicated in MS and prior to that validating the expression stability of commonly used housekeeping genes for their use in future gene expression experiments.

We selected some frequently used housekeeping genes from literature to determine their expression stability in our experimental setting. MS is a major cause of non-traumatic neurological disability deemed to affect more than 2 million people worldwide (Blight, 2011). It manifests as a chronic inflammation in central nervous system (CNS) that leads to demyelination and neurodegeneration. The disease typically manifests at 20–40 years of age when people are in their full employment and sometimes develops into an aggressive stage that alters the lives of patients and their families. Unfortunately the current treatments are only effective in preventing relapses and slowing down progression but not completely ceasing it. Although the pathogenesis of MS is not well understood, accumulating evidence suggests a complex interplay of both genetic and environmental factors (Al-Bader and Al-Sarraf, 2005; Compston and Coles, 2008; Oksenberg et al., 2008). A plethora of gene expression studies have been undertaken in peripheral mononuclear white blood cells (Der et al., 1998; Ramanathan et al., 2001; Wandinger et al., 2001; Bomprezzi et al., 2003; Koike et al., 2003; Sturzebecher et al., 2003; Hong et al., 2004; Iglesias et al., 2004; Satoh et al., 2006), in MS brain tissues (Becker et al., 1997; Whitney et al., 1999; Chabas et al., 2001; Whitney et al., 2001; Lock et al., 2002; Mycko et al., 2003; Tajouri et al., 2003; Lindberg et al., 2004; Mycko et al., 2004) and in CSF (Brynedal et al., 2010). Proteomic approaches have also been used to identify differentially expressed proteins in the CSF of MS patients (Dumont et al., 2004; Hammack et al., 2004; Noben et al., 2006). However, the proteomics analysis of CSF obtained from MS patient is relatively challenging. Since proteins are highly abundant, diversified, and soluble, only some protein subgroups may be detected and others important proteins may fail to be identified by proteomics approach. Thus, it would be prudent to use proteomic analysis along with other approaches such as gene expression profiling using microarray. Another similar but totally distinct neurological disease known as NMO shares many pathological similarities with MS and therefore it was previously considered as its variant. For this reason clinicians often used to encounter difficulty in distinguishing MS from NMO and hence similar treatment was provided to both the category of patients. However, recent research shows that there are some NMO specific IgG antibodies present in the sera of NMO patients, which differentiate both the diseases (Lennon et al., 2004).

In MS, axonal damage is widely accepted as the major cause of persistent functional disability, although its origin is unknown. During the relapsing-remitting disease course the patient’s brain itself is capable of repairing the damage, remyelinating the axon and recovering the neurological function. CSF is in contact with brain parenchyma (Rossi et al., 2012, 2014) and a site of deposition of cellular damaged products, which can influence the cellular physiology of brain cells. It is a promising biofluid in the search for biomarkers and disease associated proteins in MS, both with respect to inflammatory and neurodegenerative processes. Exposure of CGNs with CSF from diseased states can allow us to understand the pathophysiology of MS but prior to that evaluation of housekeeping genes to accurately normalize target genes is a crucial step. Selected housekeeping genes were quantified using real time PCR to accurately normalize target genes in our experimental setting. The expression stability of reference genes was further assessed by *GeNorm* and *NormFinder* algorithms. *GeNorm* program defines the gene stability as the average pairwise variation of a particular gene with all other control genes and ranks the genes according to their average expression stability denoted by *M* (Vandesompele et al., 2002). The gene with minimum *M* value is considered to be highly stable whereas the gene with highest *M* value is least stable and can be excluded. An alternative program, *NormFinder*, ranks the candidate reference genes based on the combined estimates of both intra- and intergroup variations (Andersen et al., 2004).

## Materials and Methods

All procedures were approved by the Committee of Animal Care of Prince Felipe Research Center (CIPF), Valencia, in accordance with the regulations of the European Union and Spanish legislation. Informed consent was obtained from all the patients and controls for this study and authorized by the Ethical Committee of the Institute.

### Patient Cohort

#### Patient Population

A total of 59 patients were recruited and CSF samples were obtained from the Department of Neurology, Hospital La Fe and Hospital Clinico, University of Valencia. Out of 59 patients, 21 had inflammatory MS (11 IgM+/+ and 10 IgM +/-), 8 had medullary subtype, 11 had PPMS, 9 had NMO, and 10 were non-inflammatory neurological controls (NIND patients). In CSF, apart from factors related to MS or NMO, there are factors from other diseases that produce their action. This must be considered as “*background noise*” as average population. Mixing of total CSF samples in all clinical forms may potentiate the factors related to MS. Therefore, we mixed CSF samples in all clinical forms.

Multiple sclerosis patients were defined and grouped in different clinical courses, according to the current criteria (Lublin and Reingold, 1996) and diagnosed according to McDonald criteria. They all met the following characteristics: oligoclonal IgG bands (OCGB) present, not in a phase of relapse, and have spent more than a month after the last dose of steroids. Wingerchuk criteria were used to diagnose patients with NMO disease (Wingerchuk et al., 2006). Patients suffered relapses of optic neuritis and myelitis, and two of the three criteria, normal MRI or that did not accomplish the Patty criteria for MRI diagnosis of MS. **Table 1** illustrates the clinical characteristics of the patients.

**Table 1:** Clinical characteristics of the patients studied.

Case #	Sex	Working clinical form	Clinical form	Age (years)	Evolution time	Actual EDSS
1	Female	RRMS (+/-)	RRMS	23	5	1.50
2	Female	RRMS (+/-)	SPMS	21	18	4.00
3	Female	RRMS (+/-)	RRMS	36	4	1.50
4	Male	RRMS (+/-)	RRMS	22	6	1.50
5	Female	RRMS (+/-)	RRMS	21	3	3.00
6	Female	RRMS (+/-)	RRMS	30	22	4.00
7	Female	RRMS (+/-)	RRMS	29	10	1.50
8	Female	RRMS (+/-)	RRMS	29	7	1.50
9	Female	RRMS (+/-)	RRMS	28	10	5.50
10	Female	RRMS (+/-)	RRMS	28	4	1.00
11	Female	RRMS (+/+)	RRMS	37	7	3.50
12	Male	RRMS (+/+)	RRMS	32	4	1.00
13	Female	RRMS (+/+)	RRMS	44	5	2.00
14	Female	RRMS (+/+)	RRMS	26	5	2.00
15	Female	RRMS (+/+)	RRMS	14	18	3.50
16	Male	RRMS (+/+)	RRMS	25	11	2.00
17	Female	RRMS (+/+)	SPMS	21	25	8.50
18	Female	RRMS (+/+)	RRMS	17	16	2.00
19	Female	RRMS (+/+)	SPMS	23	18	6.50
20	Female	RRMS (+/+)	SPMS	22	5	4.00
21	Female	RRMS (+/+)	RRMS	29	5	2.50
22	Male	MedMS	SPMS	39	10	4.50
23	Female	MedMS	SPMS	25	6	7.00
24	Female	MedMS	SPMS	25	14	8.00
25	Male	MedMS	SPMS	34	9	6.00
26	Male	MedMS	SPMS	34	6	6.50
27	Female	MedMS	RRMS	23	5	4.00
28	Female	MedMS	SPMS	40	10	7.50
29	Female	MedMS	SPMS	23	28	6.50
30	Female	PPMS	PPMS	54	12	7.00
31	Male	PPMS	PPMS	40	23	6.00
32	Female	PPMS	PPMS	52	14	5.50
33	Female	PPMS	PPMS	38	11	5.50
34	Male	PPMS	PPMS	31	24	6.00
35	Female	PPMS	PPMS	47	14	5.50
36	Male	PPMS	PPMS	49	11	6.00
37	Female	PPMS	PPMS	26	13	6.50
38	Female	PPMS	PPMS	34	6	5.00
39	Female	PPMS	PPMS	39	8	8.50
40	Male	PPMS	PPMS	18	15	8.00
41	Female	NMO	NMO	39	5	9.00
42	Female	NMO	NMO	50	4	7.00
43	Male	NMO	NMO	15	17	4.00
44	Male	NMO	NMO	42	5	3.50
45	Female	NMO	NMO	22	5	2.50
46	Female	NMO	NMO	27	5	2.00
47	Male	NMO	NMO	9	14	1.00
48	Female	NMO	NMO	8	32	4.00
49	Male	NMO	NMO	19	20	8.50
50	Male	CONTROL	CONTROL	23	NA	NA
51	Female	CONTROL	CONTROL	77	NA	NA
52	Female	CONTROL	CONTROL	33	NA	NA
53	Female	CONTROL	CONTROL	32	NA	NA
54	Male	CONTROL	CONTROL	59	NA	NA
55	Female	CONTROL	CONTROL	36	NA	NA
56	Female	CONTROL	CONTROL	57	NA	NA
57	Male	CONTROL	CONTROL	37	NA	NA
58	Female	CONTROL	CONTROL	21	NA	NA
59	Male	CONTROL	CONTROL	13	NA	NA

*EDSS, Kurtzke expanded disability status scale (method of quantifying disability in MS); MedMS, medullary MS; RRMS, relapsing-remitting multiple sclerosis; PPMS, primary progressive multiple sclerosis; NMO, neuromyelitis optica; +/-, presense of IgG but no IgM antibodies in the CSF; +/+, presense of both IgG and IgM antibodies in the CSF; NA, not applicable.*

#### Patient Characteristics

##### Inflammatory MS (RRMS and SPMS forms)

MS is categorized into: (1) Relapsing remitting MS (RRMS) that later develops into secondary progressive stage (SPMS); and (2) primary progressive MS (PPMS). Over 95% of patients with MS show oligoclonal bands (OCBs) of IgG in CSF (G+) (Kostulas et al., 1987) and 40% show IgM OCBs in CSF (M+) related to a more aggressive course of disease (Sharief and Thompson, 1991). In our project we also classified and named inflammatory MS into *“IgM+/-”* and *“IgM+/+ subtype”* (see below) on the basis of aggressivity and prognosis that is more complete than just RRMS or PPMS. In addition we have studied separately a set of patients with MS but with a predominant affectation of the spinal cord, because these patients have some peculiarities, and we wanted to explore if they have some differences in light of our experiments. The most aggressive cases termed as *“medullary”* have more spinal injuries.

###### IgM+/- clinical form of MS

Patients named as *“IgM+/*-*subtype”* had IgG antibodies (+) but no IgM (-) oligoclonal antibodies detected in the CSF of brain.

###### IgM+/+ clinical form of MS

Patients named as *“IgM+/+ subtype”* had both IgG antibodies (+) and IgM (+) oligoclonal antibodies detected in the CSF of brain.

##### Medullary clinical form of MS

All these patients were positive for OCGBs and negative for oligoclonal IgM bands (OCMBs) in CSF of spinal region. The patients accomplished the Swanton criteria for dissemination in time.

##### Primary progressive MS

These patients are characterized by progressive decline in neurological disability.

##### Neuromyelitis optica patients

Individuals diagnosed with NMO met at least two of the following three features. (1) Long extensive transverse myelitis (>3 vestibule bodies); (2) Antibodies against aquaporin-4; (3) Normal brain at the first event.

##### Controls [Non-Inflammatory Neurological Diseases (NIND)]

Individuals who were suspected to have MS but were not diagnosed with MS were classified as controls.

#### Cerebrospinal Fluid Samples of Patients

Cerebrospinal fluid samples were obtained by lumbar puncture at the time of diagnosis. Samples were centrifuged for 10 min at 700 × *g* and aliquots were frozen at -80°C until use. No patient had received treatment with immunosuppressive drugs, immunomodulators or corticosteroids for at least 1 month prior to the extraction of CSF.

#### Cerebrospinal Fluid Studies

All the studies were performed by immunologists who were blind to the clinical and MRI data.

##### Oligoclonal band studies

Paired CSF and serum samples were analyzed to detect OCBs (OCGB and OCMB) by isoelectric focusing (IEF) and immunodetection. We used a commercial kit to determine OCGB (Helena BioScience IgG-IEF Kit) and the technique described by Villar et al. (2001) to detect OCMB. Serum samples were diluted in saline before the IEF in order to reach the same concentration range as that of CSF samples. All samples were incubated with 50 mmol/L dithiothreitol at pH 9.5 to reduce IgM. Focusing was performed on a Multiphor II Electrophoresis System (GE Healthcare) at pH 5–8. Proteins were then transferred to a PVDF membrane and analyzed by Western blot. Finally, immunodetection was performed by biotin-conjugate-goat anti-human IgM and streptavidin-alkaline phosphatase (Sigma–Aldrich).

##### Serum studies

**FIGURE 1:**
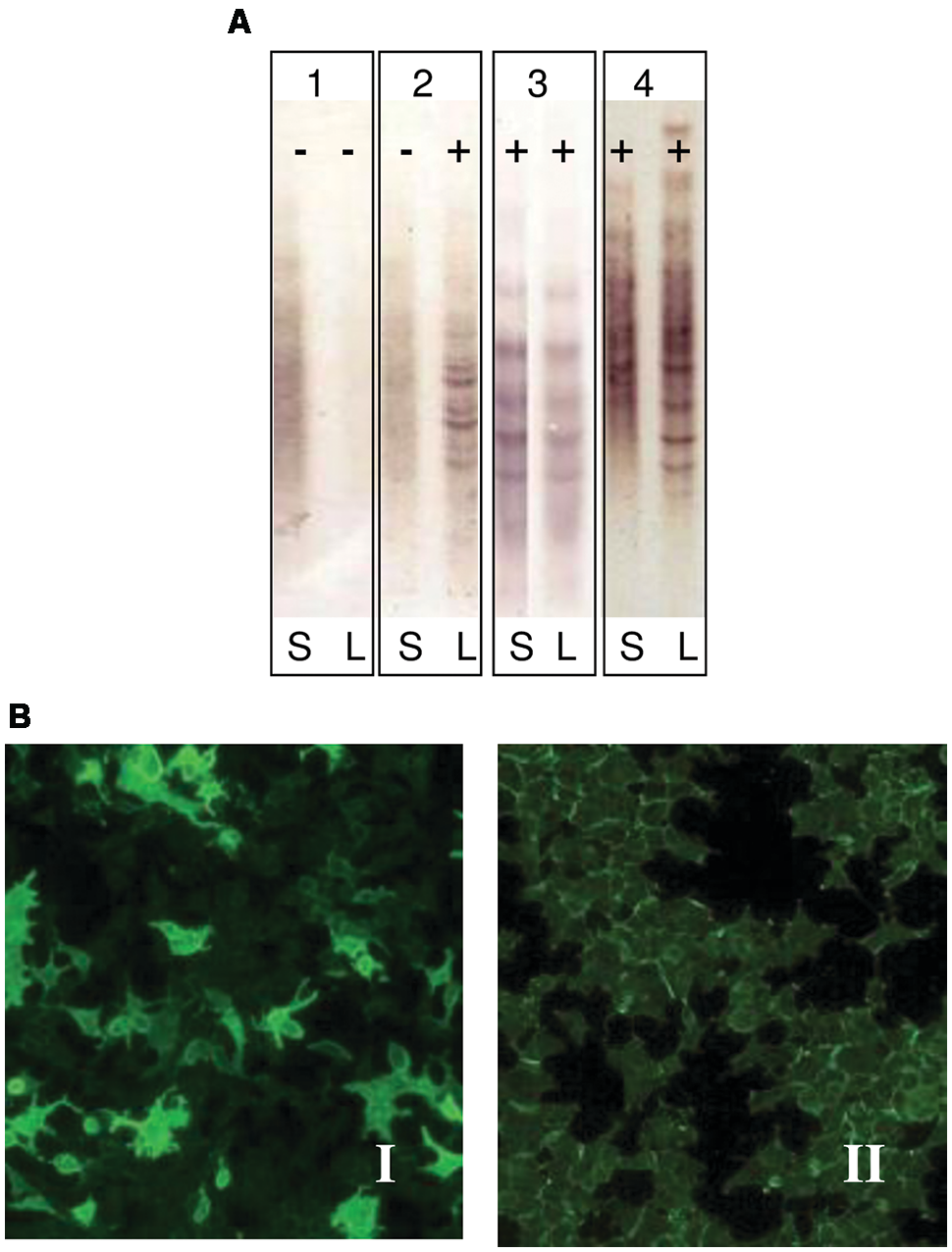
**(A)** Immunodetection of oligoclonal bands (OCBs) in serum (S) and cerebrospinal fluid (CSF) (L). Pattern 1: No OCBs seen (negative, polyclonal): No OCBs in CSF or serum. No intrathecal Ig synthesis; Pattern 2: OCBs in CSF only (positive): OCBs present in CSF only. Intrathecal IgG synthesis as seen in MS; Pattern 3: Identical OCBs in both (mirror images): Bands in serum mirror those in CSF. This suggests systemic Ig synthesis; Pattern 4: Identical OCBs in both with extra bands in CSF: Identical bands in both serum and CSF with extra bands in CSF. Image demonstrates both intrathecal and systemic Ig synthesis. This is identical as it is seen in MS. **(B)** Indirect immunofluorescence in cells transfected by aquaporin 4 (EUROIMMUN Aquaporin-4 IIFT). Panel I: Anti-AQP4 antibodies observed in the serum of NMO patients (positive sample); Panel II: Absence of anti-AQP4 antibodies in serum sample (negative sample).

Anti-AQP4 antibody in NMO has a high specificity so as to contribute to early diagnosis and optimized treatment of Devic disease. Serum sample diluted 1:10 in PBS-Tween was used to detect the presence of NMO specific IgG antibodies. Indirect immunofluorescence (IFI) was performed to diagnose NMO (**Figure 1B**). Antibodies against aquaporin 4 were detected using a cell line, which was molecular biologically modified to produce large quantities of aquaporin 4. In this method (EuroImmun IIFT) recombinantly transfected cells act as an antigen substrate to be incubated with diluted serum samples for half an hour.

### Animals

Wistar rats (Harlan Iberica) with weight between 200 and 250 g were used. All animals were raised under controlled conditions with cycles of light/dark (12/12 h), temperature of 23°C and humidity of 60%. Access to water and food (standard rodent feed supplied by Harlan, Teklad 2014 Global 14% Protein Rodent Maintenance Diet) was provided. To obtain offspring, pregnant females were separated and kept in isolated cages during gestation. The maintenance of the animals was performed in the animal facilities unit of Prince Felipe Research Center, Valencia, Spain.

### Primary Culture of Cerebellar Granule Neurons

All operations were performed under sterile conditions in vertical laminar flow chamber (Telstar AV-100 and Bio-II-A). The cells were kept in an incubator at 37°C in a humidified atmosphere composed of 95% air and 5% CO_2_ (CO2 incubator Thermo Form, model 371). Primary cultures of CGNs were obtained according to previously described modified protocol (Minana et al., 1998). Forebrains were collected from 8 days old Wistar rats, mechanically dissociated and cerebellum was dissected. Isolated cerebella were stripped of meninges, minced by mild trituration with a Pasteur pipette and treated with 3 mg/ml dispase (grade II) for 30 min at 37°C in a 5% CO_2_ humidified atmosphere. After half an hour, dispase was inactivated with 1mM EDTA. Granule cells were then resuspended in basal Eagleś medium (BME, Gibco, ref. 41010) with 40 μg/ml of DNaseI. The cell suspension was filtered through a mesh with a pore size of 90 μm and centrifuged at 1500 rpm for 5 min and thereafter, cell suspension was washed three times with BME. Finally, the cells were resuspended in complete BME medium with Earleś salts containing 10% heat inactivated FBS (fetal bovine serum, Gibco), 2 mM glutamine, 0.1 mg/ml gentamycin and 25 mM KCl. The neuronal cells were counted and plated onto poly-L-lysine coated 6-well (35-mm) culture dishes (Fisher) at a density of 3 × 10^5^ cells/well and incubated at 37°C in a 5% CO_2_/95% humidity atmosphere. After 20 min at 37°C, the medium was removed and fresh complete medium was added. Since the purpose of our study was to obtain pure cultures of CGNs, it was necessary to add a chemical that can prevent the growth of non-neuronal cells. Twenty micro liter of cytosine arabinoside (1 mM) was added to each culture plate after 18–24 h to inhibit replication of non-neuronal cells. The cells were kept in an incubator at 37°C in a humidified atmosphere composed of 95% air and 5% CO_2_ (CO_2_ incubator Thermo Form, model 371). Cells were fed every 3–4 days in culture with 5.6 mM glucose.

**FIGURE 2:**
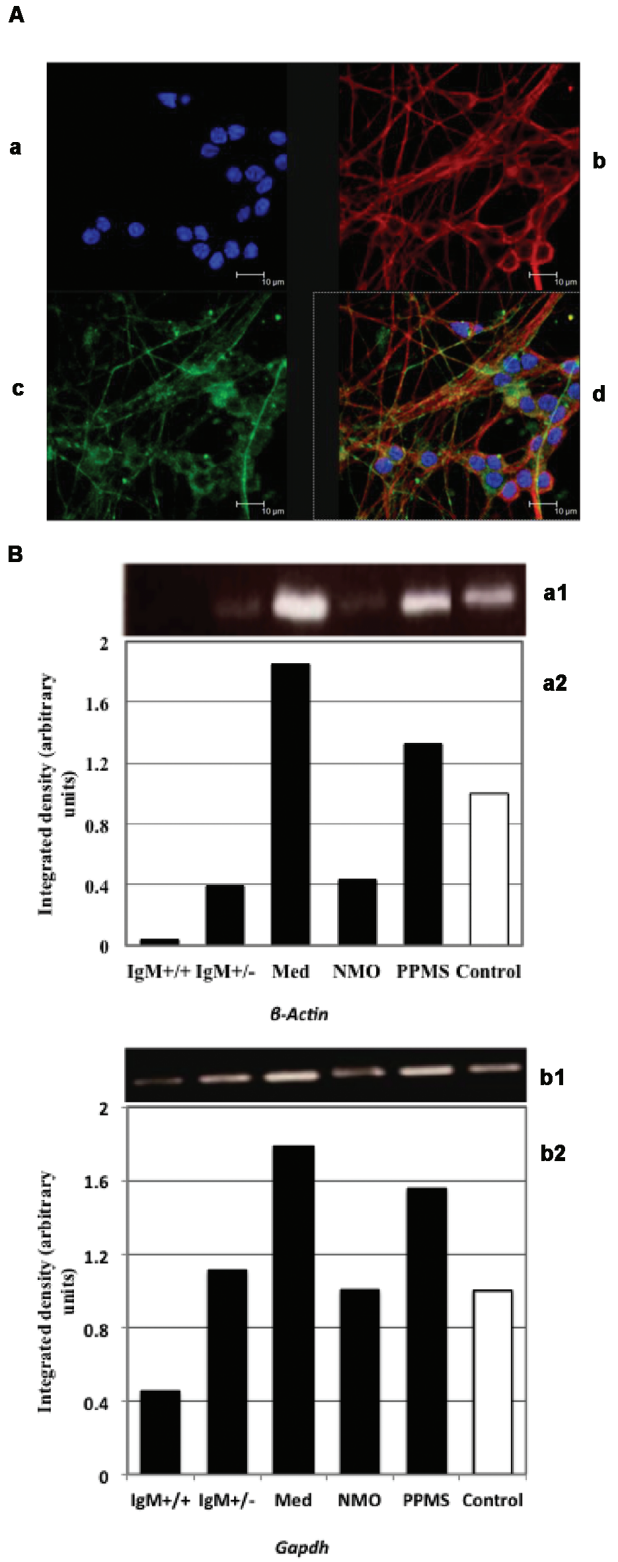
**(A)** Immunofluorescence of primary cultures of CGNs. (a) DAPI stained nuclei. Neurofilaments stained with (b) Texas red and (c) FITC dyes. (d) Merged images. **(B)** Analysis of *β-actin* (a) and *Gapdh* (b) expression by PCR: (1) Electrophoretic bands; (2) Integrated density obtained by Image J software quantification. The values correspond with the fold change *vs*. control values of the MS clinical forms, NMO, and control patients.

Cerebellar granule neurons were stained with Texas Red and FITC dyes. The nuclei of neurofilaments were stained with DAPI. **Figure 2A** shows pure cultures of granule neurons isolated from cerebellum with stained neurofilaments.

### Confocal Microscopy

The living cells were always kept at 37°C and 5% CO_2_. Cells were analyzed on a Leica TCS SP2 confocal microscope AOBS (Leica Microsystems) inverted laser scanning confocal microscope using a 63 × Plan-Apochromat-Lambda Blue 1.4 N.A. oil objective lens. All confocal images were obtained under identical scan settings. Images of 1,024° × 1,024 pixels, 8-bits were collected for each preparation. Best focus was based on highest pixel intensity. Imaging conditions were identical for all the images, and no images were saturated. Metamorph 7.0 (Molecular Devices, Downingtown, PA, USA) was used for image analysis on the images collected.

### Selection of Housekeeping Genes

Candidate housekeeping genes were selected from those most commonly used in literature including β-actin (*ActB*), hypoxanthine guanine phosphoribosyl-transferase (*Hprt*), ribosomal protein L19 (*Rpl19*), lactate dehydrogenaseA (*Ldha*), transferrin receptor (*Tfrc*), microglobulin beta-2 (*B2m*), and glyceraldehyde-3-phosphate-dehydrogenase (*Gapdh*). The function and references of the genes are listed in **Table 2**. The primers for the selected reference genes from 5′- to 3′- end were as follows: *Actb* forward ATTGAACACGGCATTGTCAC, reverse ACCCTCATAGATGGGCACAG; *Hprt* forward CCTCTCGAAGTGTTGGATACAG, reverse TCAAATCCCTGAAGTGCTCAT; *Rpl19* forward ACCTGGATGCGAAGGATGAG, reverse CCATGAGAATCCGCTTGTTT; *Ldha* forward AGGAGCAGTGGAAGGATGTG, reverse AGGATACATGGGACGCTGAG; *Tfrc* forward GTTGTTGAGGCAGACCTTCA, reverse ATGACTGAGATGGCGGAAAC; *B2m* forward GTCGTGCTTGCCATTCAGA, reverse ATTTGAGGTGGGTGGAACTG; *Gapdh* forward GGAAACCCATCACCATCTTC, reverse GTGGTTCACACCCATCACAA.

**Table 2:** Panel of seven candidate housekeeping genes selected for expression analysis.

Gene symbol	Gene name	mRNA accession number	Function	Reference
*ActB*	β-Actin	NM_031144	Cytoskeletal structural Protein	Stürzenbaum and Kille, 2001
*Hprt*	Hypoxanthine guanine phosphoribosyl transferase	NM_012583	Metabolic salvage of purines	Everaert et al., 2011
*Rpl19*	Ribosomal protein L19	NM_031103	Unclear	Zhou et al., 2010
*Ldha*	Lactate dehydrogenase A	NM_017025	NADH dependent enzyme that catalyzes reduction of pyruvate to lactate	–
*Tfrc*	Transferrin Receptor	NM_022712	Iron delivery from transferrin to cells	Gorzelniak et al., 2001
*B2m*	Microglobulin-b-2	NM_012512	Major histocompatibility complex class I	Yurube et al., 2011
*Gapdh*	Glyceraldehyde-3- phosphate-dehydrogenase	NM_017008	NAD+ dependent enzyme that catalyzes conversion of glyceraldehyde-3-phosphate to 1,3-bis phosphoglycerate	Fort et al., 1985; Harrison et al., 2000; Medhurst et al., 2000; Gorzelniak et al., 2001

### Treatment, Total RNA Isolation and cDNA Synthesis

Neuronal cell cultures were incubated with 10% v/v CSF from MS (IgM+/-, IgM+/+, medullary, PPMS) patients, NMO patients and controls for 24 h. This step was performed to identify transcriptional changes in future transcriptomic experiments. Total RNA was isolated from cell cultures exposed to the CSF of different experimental conditions (IgM+/-, IgM+/+, medullary, NMO, PPMS, Control) using Quick RNA MicroPrep Kit (Zymo Research Corp.). The RNA concentration was determined spectrophotometrically at 260 nm using the Nanodrop 1000 spectrophotometer (V3.7 software) and RNA purity was checked by means of the absorbance ratio at 260/280 nm. Isolated RNA was stored at -80° and later reverse transcribed to cDNA. The cDNA was synthesized and stored at -20°C. Primers for selected genes were designed using Primer 3 software. PCR was performed in a thermocycler (BioRad) with cycling conditions (94° for 30 s, 40 cycles at 59° for 30 s and 72° for 30 s). Each 25 μl reaction contained 12.5 μl Master Mix (Applied Biosystems), 1 μl gene-specific forward and reverse primers (0.5 μM), 1 μl undiluted cDNA and 10.5 μl DEPC (nuclease free) treated water. Negative controls with no template contained nuclease-free water instead.

### Agarose Gel Electrophoresis and Real-time Polymerase Chain Reaction of Selected Housekeeping Genes

The electrophoresis was performed in 1.5% agarose gels, and they were run at 50 V, stained with ethidium bromide, photographed and evaluated with ImageJ software. A DNA ladder control (100 bp, Invitrogen) was also used in the electrophoresis to evaluate DNA fragment size. Real time PCR was performed in a 96-well plate (Roche) incubated in thermocycler (LC480, Roche) with cycling conditions (94° for 15 s, 45 cycles at 60° for 30 s and 72° for 30 s). Each 10ml reaction contained 5 ml SYBR Green Master Mix (Applied Biosystems), 1 μl gene-specific forward and reverse primers (0.5 μM), 1 μl undiluted cDNA and 3 ml DEPC (nuclease free) treated water. Negative controls with no template contained nuclease-free water instead. All samples were run in duplicate and average values were calculated. Data was analyzed using 7300 Sequence Detection Software (SDS) Version 1.3 (Software Roche). Following qRT-PCR, a dissociation curve was run to check the PCR product specificity.

### Determination of Reference Gene Expression Stability

To determine the stability of these genes on the basis of their Cp values, we employed comparative ΔCT method. Data are plotted as fold change values which were calculated by 2^Δ(Ctexp -Ctcontrol)^. Cp value is defined as the PCR cycle at which the fluorescent signal of the reporter dye crosses an arbitrarily placed threshold. Invariable genes were later assessed by publicly available software tools named *GeNorm* and *NormFinder.*

## Results

### Demographic and Clinical Profiles of MS, NMO and NIND Groups

Patients were classified according to detection of OCBs (**Figure 1A**) and of aquaporin antibodies (**Figure 1B**). Baseline characteristics of the study population are described in **Table 3**. Prevalence of MS was found more in women (75%) than in men. Mean age of MS patients was 30.7 ± 9.7 whereas 25.6 ± 15 for NMO patients. According to the clinical classification, the general characteristics of MS patients are described in **Table 4**. There were significant differences observed between the age at beginning of PPMS and the other two MS forms (*p* < 0.003); between the EDSS of RRMS and the two other MS forms (<0.001); the evolution time between PPMS and RRMS (*p* = 0.043) after Bonferroni correction. **Table 5** shows the characteristics of MS patients according to new proposal and working classification. After Bonferroni correction, significance was due to differences between the age at beginning and the EDSS between medullary MS and PPMS with the inflammatory MS.

**Table 3:** General characteristics of series studied.

	Controls (*n* = 10)	MS patients (*n* = 40)	NMO patients (*n* = 9)	*p*
% Females (*n*)	60.0 (6)	75.0 (30)	55.6 (5)	0.40 (χ^2^)
Age (mean, *SD*)	40.3 (19.5)	30.7 (9.7)	25.6 (15.0)	0.04 (ANOVA test)
EDSS	n.a.	4.5 (2.3)	4.6 (2.8)	0.94 (*t*-test)
Evolution time	n.a.	11.1 (6.6)	11.8 (9.7)	0.79 (*t*-test)

**Table 4:** Characteristics of MS patients according to the clinical classification.

	RRMS (*n* = 18)	SPMS (*n* = 11)	PPMS (*n* = 11)	*p*
% Females (n)	83.3 (15)	72.7 (8)	63.6 (7)	0.48 (χ^2^)
Age (mean, *SD*)	27.3 (7.2)	27.9 (7.3)	38.9 (11.2)	0.003 (ANOVA test)
EDSS	2.4 (1.2)	6.2 (1.5)	6.3 (1.1)	<0.001 (ANOVA test)
Evolution time	8.1 (5.4)	13.5 (7.8)	13.8 (5.7)	0.043 (ANOVA test)

*After Bonferroni correction, significance were due to differences between the age at beginning between PPMS and the other two MS forms; between the EDSS of RRMS and the two other MS forms; the evolution time between PPMS and RRMS.*

**Table 5:** Characteristics of MS patients according to new proposal and working classification.

	Inflammatory MS (*n* = 21)		Medullar MS (*n* = 8)	PPMS (*n* = 11)	*p*
	G+/M- (*n* = 10)	G+/M+ (*n* = 11)			
% Females (*n*)	90 (9)	81.8 (9)	62.5 (5)	63.6 (7)	0.40 (χ^2^)
Age (mean, *SD*)	26.7 (4.8)	26.3 (8.7)	31.4 (7.0)	38.9 (11.2)	0.005
EDSS	2.5 (1.5)	3.4 (2.2)	6.2 (1.4)	6.3 (1.1)	0.000
Evolution time	8.9 (6.3)	10.8 (5.9)	8.5 (3.1)	13.8 (5.7)	0.154

*After Bonferroni correction, significance was due to differences between the age at beginning and the EDSS between Medullar MS and PPMS with the inflammatory MS*.

We found significant differences in the age at beginning between PPMS and the other two MS forms (RRMS and SPMS) after Bonferroni correction (*p* < 0.003) (**Table 6**). People with PPMS are usually older at the time of diagnosis with an average age of 40. Furthermore, different subtypes of MS help predict disease severity and response to treatment hence their categorization is important. In our study, we found significant differences between the “*Expanded Disability Status Scale*” (EDSS) of RRMS and the two other MS forms (SPMS and PPMS) (*p* < 0.001) (**Table 5**). Although nerve injury always occurs, the pattern is specific for each individual with MS. Disease severity and disability increases from relapsing-remitting to secondary progressive course and in PPMS subtype, symptoms continually worsen from the time of diagnosis rather than having well-defined attacks and recovery. PPMS usually results in disability earlier than relapsing-remitting MS. Significant differences were found in the evolution time from the first to the second episode between RRMS and PPMS (*p* = 0.043). In patients experiencing a progressive course, evolution time was similar in secondary progressive cases and in cases that were progressive from onset (13.5 versus 13.8) (**Table 5**).

**Table 6:** Candidate housekeeping genes ranked in cerebellar granule neurons treated with CSF of MS/NMO patients according to their expression stability by *Genorm* and *Normfinder* methods.

*Genorm*			*Normfinder*		
Ranking order	Gene name	Average *M* value	Ranking order	Gene name	Stability value
**1**	***Tfrc***	**1.092**	**1**	***Tfrc***	**0.546**
**1**	***B2m***	**1.092**	**2**	***Ldha***	**0.589**
**2**	***Rpl19***	**1.198**	**3**	***Rpl19***	**0.972**
3	*Ldha*	1.253	4	*B2m*	1.102
4	*Hprt*	1.318	5	*Hprt*	1.379
5	*ActB*	2.929	6	*ActB*	6.099
6	*Gapdh*	4.201	7	*Gapdh*	6.953

*M, average expression stability measure of seven reference genes with Tfrc the most stable gene and Gapdh the least stable. Lower M value of average expression stability indicates more stable expression while the highest M value indicates variable expression. Bold indicates most stably expressed genes.*

According to the new proposal and working classification, inflammatory MS subtypes shared similar age at disease onset (mean = 26.7 versus 26.3 years; *p* = 0.005). Significant differences were found between the age at disease onset in medullary MS and PPMS with the inflammatory MS (*p* < 0.005). The degree of disability as measured by EDSS was similar in medullary MS and PPMS (6.2 versus 6.3) whereas significant differences were found between disability extent in medullary MS and PPMS with the inflammatory MS (*p* < 0.001). IgM+/- represents the less aggressive inflammatory subtype with OCGB in CSF with poor prognosis whereas IgM+/+ signifies a more aggressive category with OCGB and OCMB in CSF with worse prognosis. On the contrary, medullary MS represents the most aggressive subtype of MS with increased neurological disability and dysfunction as compared to inflammatory subtypes. Disability in patients experiencing PPMS worsens over time with no relapses and remission.

### Identification of Stably Expressed Housekeeping Genes

#### PCR of *Gapdh* and *β-actin*

We first quantified *ActB* and *Gapdh* genes using conventional PCR in treated neuronal samples and ran agarose gel electrophoresis. We found that both *β-Actin* and *Gapdh* genes, which are presumed to express at constant levels showed varying band intensity in CGNs when treated with the CSF from MS and NMO patients (**Figure 2B**). From this data we conclude that both *ActB* and *Gapdh* genes are not suitable to normalize gene transcripts in our experimental conditions.

#### Quantitative PCR of Housekeeping Genes in our Experimental Conditions

Quantitative real time PCR was performed for a group of frequently used reference genes. *GeNorm* and *NormFinder* algorithms were used to assess the most stably expressed genes. Our data suggests *Tfrc, B2m* as the most stable genes followed by *Rpl19* using *GeNorm* software (Average expression stability value denoted by M: 1.09 for *Tfrc* and *B2m;* M: 1.19 for *Rpl19*). Similarly, *Tfrc* showed most stable expression as assessed by *NormFinder* algorithm followed by *Ldha* and *Rpl19* (M: 0.54 for *Tfrc*; M: 0.58 for *Ldha* and 0.97 for *Rpl19*) (**Table 6**). On the other hand, *β-Actin* and *Gapdh* showed highest fluctuation in our experimental conditions with 2.9 and 4.2 as the average expression stability value by *GeNorm*. Therefore their use is strictly discouraged while normalizing gene expression data in studies related to the current one.

**FIGURE 3:**
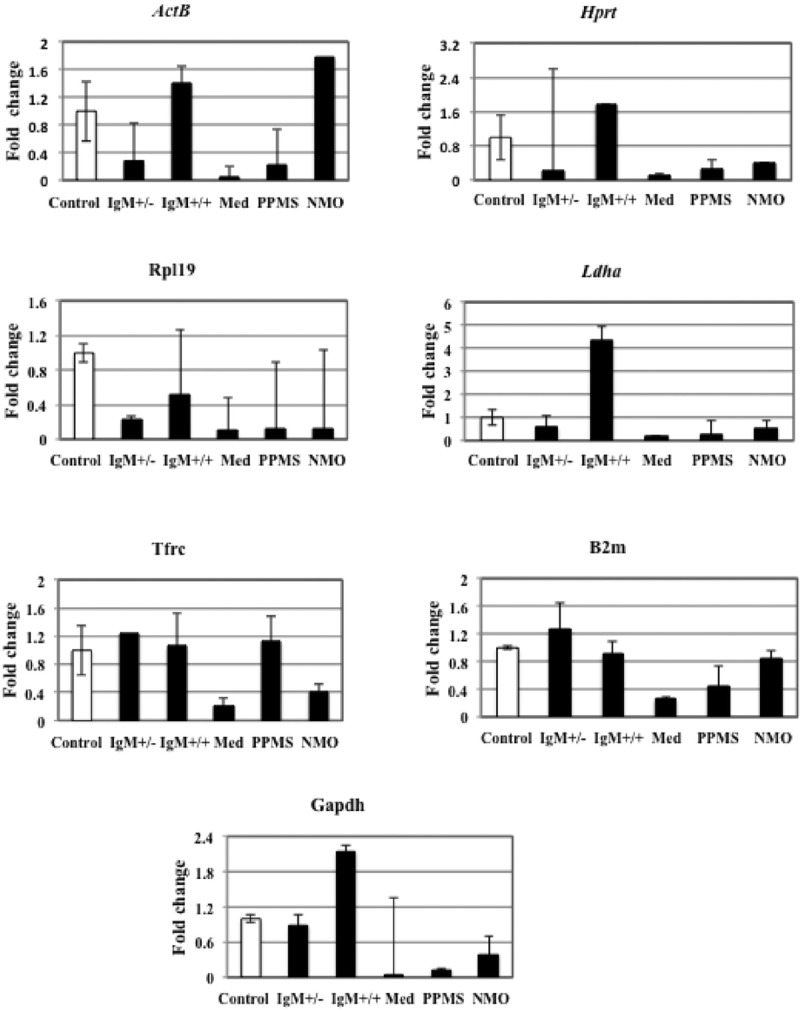
**Fold change for each reference gene tested in distinct disease courses of multiple sclerosis (MS).** IgM+/+ and IgM+/-: Inflammatory forms of relapsing remitting MS; Med: medullary form; NMO: neuromyelitis optica; Control: other non-inflammatory neurological diseases (NIND).

**Table 6** illustrates candidate housekeeping genes ranked in CGNs treated with CSF from MS/NMO patients according to their expression stability by *GeNorm* and *NormFinder* methods. The C_t_ values of all the experimental conditions obtained from qPCR experiment were normalized to control. Then we plotted the fold change values for each reference gene tested in distinct disease courses of MS and NMO patients (**Figure 3**). Fold change was calculated by 2^Δ(Ctexp -Ctcontrol)^.

##### ActB

We found that the expression of *ActB* gene dropped to 0.2 folds in neurons treated with IgM+/- MS patients and increased again to 1.4 folds in IgM+/+ treated neurons, as compared to control. In neurons treated with medullary CSF the gene expression dropped to 0.04 folds and 0.2 folds in PPMS and increased to 1.78 folds in NMO patients compared to control. Although the variation in the expression level of this gene in all the different experimental conditions is not large as seen by qPCR data, we employed *GeNorm* software to compare the expression stability of all the reference genes with each other and identify the best reference gene out of a group of commonly used reference genes to avoid getting biased results. The software *GeNorm* ranked *ActB* gene as second last unstable gene as compared to the expression levels of other selected reference genes (M value: 2.92 using *GeNorm*). We conclude that this gene varies in our experimental conditions with respect to other selected reference genes. Hence it should not be used to normalize gene expression data in our experimental conditions.

##### Hprt

The data indicates that *Hprt* gene was 0.23 folds downregulated in neurons treated with IgM+/- CSF of MS patients as compared to control. The expression was up regulated 1.7 times in IgM+/+ treated neurons and again down regulated by 0.1 folds, 0.27 folds and 0.4 folds in neurons treated with the CSF of medullary, PPMS and NMO patients as compared to control. According to *GeNorm* program, *Hprt* was ranked third last unstable reference genes with respect to other reference genes (Average expression stability value: 1.3).

##### Rpl19

*Rpl19* gene expression was down regulated by 0.2 and 0.5 folds in IgM+/- and IgM+/+ treated neurons as compared to control. There was only 0.1 folds decrease in *Rpl19* gene expression when neurons were treated with medullary, PPMS and NMO patients as compared to control. Hence, there is a negligible variation in all the experimental conditions as indicated by qPCR data. In agreement with this data *GeNorm* identifies this gene as the third most stable gene (M value: 1.19).

##### Ldha

There was 0.5 folds downregulation of *Ldha* gene in neurons treated with the CSF of IgM+/- patients with respect to control. The expression level increased up to fourfolds in neurons treated with IgM+/+ treated neurons. In medullary, there was a 0.17 folds decrease in gene expression and we found 0.28 folds and 0.54 folds decrease gene expression in PPMS and NMO patients. The data signifies that the expression of this gene is not constant in all the experimental conditions. The average expression stability (M) value of this gene was 1.25 and was ranked as the fourth stable gene according to *GeNorm* software.

##### Tfrc

*Tfrc* gene was up regulated by 1.2 folds in neurons treated with IgM+/- treated neurons as compared to control. In IgM+/+ treated neurons the expression level almost remained the same as compared to control. In medullary patients the expression was reduced by 0.2 folds and in PPMS treated neurons the level was increased by only 1.1 folds which was almost similar as compared to control. There was a downregulation of this gene by 0.4 folds in neurons treated with the CSF of MS patients. Overall, there was a negligible variation in the gene expression in different experimental conditions. According to the *GeNorm* algorithm the average expression stability value was 1.092 and it was ranked the best reference gene with respect to others.

##### B2m

The data indicates that there was 1.2 folds up regulation of *B2m* gene in IgM+/- treated neurons as compared to control. The expression was down regulated by 0.9 folds in IgM+/+ treated neurons which was not a large variation as compared to control. It dropped to 0.2 folds in medullary treated neurons and 0.4 folds in PPMS treated neurons. The expression decreased by 0.8 folds in NMO treated neurons as compared to control. According to the *GeNorm* algorithm the average expression stability value was similar to *Tfrc* average expression stability (M: 1.092) and it was also ranked the best reference gene with respect to others. We conclude that both *Tfrc* and *B2m* with similar average expression stability values should be used to normalize gene expression data in our experimental conditions.

##### Gapdh

The expression level of *Gapdh* gene was 0.89 folds lower in IgM+/- treated neurons as compared to control. The expression level increased by twofolds in IgM+/+ treated neuons as compared to control. In neurons treated with the CSF of medullary MS patients the gene downregulated by 0.02 folds and by 0.1 fold in neurons treated with the CSF of PPMS patients. Similarly the expression level declined by 0.38 folds in NMO treated patients. According to qPCR data that there is a huge fluctuation in *Gapdh* gene expression in our experimental conditions. Normally, *Gapdh* is used as a housekeeping gene but we find that it is not a housekeeping gene in our experimental conditions. *GeNorm* ranked this gene as the least stable gene with 4.2 as the average expression stability value.

## Discussion

Quantitative RT-PCR has recently become the most widely accepted method of quantification for its sensitive, accurate and reliable determination of gene expression levels in cells and tissues. To avoid sample-to-sample variation, normalization of gene transcripts is required. The conventional way to perform normalization is to select a housekeeping gene whose expression is believed to remain stable in all cell types/tissues, during cellular development and under various experimental conditions then relate the expression of gene of interest to that of a housekeeping gene. For many years it has been assumed that the genes such as *β-Actin* and *Gapdh* express constitutively in all cells and tissues. *β-Actin* (*ActB*) is a cytoskeletal protein that maintains the structure and integrity of cells. GADPH, on the other hand, is a key glycolytic enzyme involved mainly in the production of energy. Since both *ActB* and *Gapdh* are involved in maintaining the basic metabolic functions of a cell, they are presumed to express at stable levels. Therefore, they are employed as common internal controls in most of the laboratories. However, several lines of evidence show that their rate of transcription is affected by a variety of factors such as epidermal growth factor, transforming growth factor-β and platelet-derived growth factor while constitutively expressed (Elder et al., 1984; Leof et al., 1986; Keski-Oja et al., 1988). Therefore, their expression may not necessarily be constant in all conditions. Furthermore, GADPH is implicated in non-metabolic processes independent of its metabolic function, such as transcription activation, vesicle transport from endoplasmic reticulum to Golgi apparatus and polymerization of tubulin into microtubules (Kumagai and Sakai, 1983; Durrieu et al., 1987; Muronetz et al., 1994; Zheng et al., 2003; Tisdale and Artalejo, 2007). Previous literature reveals that neuronal apoptosis is associated with suppressed glycolytic activity of GADPH (Burke, 1983; Dastoor and Dreyer, 2001; Makhina et al., 2009). It has been observed that GADPH interacts with other proteins which results in reduced glycolytic activity (Hara et al., 2005). This process may lead to neuroaxonal damage in neurodegenerative diseases such as Huntington’s, Parkinson’s, and Alzheimer’s disease (Vécsei and Pál, 1993; Mazzola and Sirover, 2003; Senatorov et al., 2003; Li et al., 2004; Tsuchiya et al., 2005; Kolln et al., 2010). The realization that these reference genes may fluctuate in different experiments has led to their pre-validation for their expression stability.

This is the first study to the best of our knowledge that reports the most stable HK genes in CGNs treated with CSF from MS/NMO patients. Seven commonly used housekeeping genes were chosen from the available literature. Expression levels of HK genes in different MS clinical forms were quantified by qRT-PCR. Our results reveal that *Gapdh* expression levels changed in all forms (RRMS, PPMS, NMO) as compared to controls. This gene was not among the best reference genes therefore it is strongly advised not to employ it as a control in studies related to current one. Moreover, *β-Actin*, that is often used as a loading control also showed unstable expression in all conditions, though to a lesser extent than *Gapdh.* Transferrin receptor (*Tfrc*) gene was up regulated by 1.2 folds in neurons treated with IgM+/- treated neurons as compared to control. In IgM+/+ treated neurons the expression level almost remained the same as compared to control. In medullary patients the expression was reduced by 0.2 folds and in PPMS treated neurons the level was increased by only 1.1 folds which was almost similar as compared to control. There was a downregulation of this gene by 0.4 folds in neurons treated with the CSF of MS patients. Overall, we see from the data that there was a negligible variation in the *Tfrc* gene expression in different experimental conditions. *Tfrc* is required for iron delivery from transferrin to cells. Microglobulin beta-2 (*B2m*), a component of MHC class I molecule, showed higher expression in IgM+/- treated neurons as compared to control. The expression was down regulated by 0.9 folds in IgM+/+ treated neurons which was not a large variation as compared to control. It dropped to 0.2 folds in medullary treated neurons and 0.4 folds in PPMS treated neurons. The expression decreased by 0.8 folds in NMO treated neurons as compared to control. According to the *GeNorm* algorithm the average expression stability value was similar to *Tfrc* average expression stability (M: 1.092) and it was also ranked the best reference gene with respect to others. We conclude that both *Tfrc* and *B2m* with similar average expression stability values should be used to normalize gene expression data in our experimental conditions. Hypoxanthine guanine phosphoribosyl-transferase (*Hprt*) gene showed fluctuated expression level in different experimental conditions. It plays an important role in purine salvage pathway. According to *GeNorm* program, *Hprt* was ranked third last unstable reference genes with respect to other reference genes (Average expression stability value: 1.3). Ribosomal protein L19 (*Rpl19*) showed negligible down regulation in all the different experimental conditions. *GeNorm* identifies this gene as the third most stable gene (*M* value: 1.19). On the contrary, *Ldha* gene was upregulated in IgM+/+ but down regulated in IgM+/- and medullar clinical form of RRMS. Its expression further lowered in PPMS and NMO. The data signifies that the expression of this gene was not constant in all the experimental conditions and not suitable for normalization of gene transcripts in studies related to the current one.

Overall, *geNorm* and *NormFinder* algorithms identified *Tfrc* and *B2m* the best housekeeping genes and *Gapdh* and *ActB* the most unsuitable genes in our experimental model of MS and therefore the current study demonstrates the necessity for pre-validation of HK genes for any experimental system. Since both the algorithms are based on different mathematical approaches, the order of genes was not exactly similar. However, both *geNorm* and *NormFinder* rank the traditional reference genes GAPDH and β-actin as most unstable genes. Therefore, we strongly advise to check the expression stability of these genes before using them for normalization purposes.

We conclude from data provided in this study that transferrin receptor (*Tfrc*) and microglobulin beta-2 (*B2m*) as the most stably expressed housekeeping genes in CGNs treated with CSF of MS patients. On the other hand, *Gapdh* and *β-actin* showed highly fluctuated expression indicating their unsuitability for such studies. This study demonstrates the usefulness of pre-validating the expression stability of housekeeping genes for normalization of target gene transcripts in gene expression studies. Our data suggest that it is required to determine the suitability of any common HK genes to be used for normalization in “*Omic*” studies, and even such pre-selection should be a routine step for any experimental system in a laboratory.

## Conflict of Interest Statement

The authors declare that the research was conducted in the absence of any commercial or financial relationships that could be construed as a potential conflict of interest.
